# Methylene blue induces macroautophagy through 5′ adenosine monophosphate-activated protein kinase pathway to protect neurons from serum deprivation

**DOI:** 10.3389/fncel.2013.00056

**Published:** 2013-05-03

**Authors:** Luokun Xie, Wenjun Li, Ali Winters, Fang Yuan, Kunlin Jin, Shaohua Yang

**Affiliations:** ^1^Department of Pharmacology and Neuroscience, Institute for Alzheimer’s Disease and Aging Research, University of North Texas Health Science Center at FortWorthFortWorth, TX, USA; ^2^Department of Neurosurgery, Beijing Neurosurgical Institute, Beijing Tiantan Hospital, Capital Medical UniversityBeijing, China

**Keywords:** methylene blue, AMPK, neuroprotection, Alzheimer disease, macroautophagy

## Abstract

Methylene blue has been shown to be neuroprotective in multiple experimental neurodegenerative disease models. However, the mechanisms underlying the neuroprotective effects have not been fully elucidated. Previous studies have shown that macroautophagy has multiple beneficial roles for maintaining normal cellular homeostasis and that induction of macroautophagy after myocardial ischemia is protective. In the present study we demonstrated that methylene blue could protect HT22 hippocampal cell death induced by serum deprivation, companied by induction of macroautophagy. We also found that methylene blue-mediated neuroprotection was abolished by macroautophagy inhibition. Interestingly, 5′ adenosine monophosphate-activated protein kinase (AMPK) signaling, but not inhibition of mammalian target of rapamycin signaling, was activated at 12 and 24 h after methylene blue treatment in a dose-dependent manner. Methylene blue-induced macroautophagy was blocked by AMPK inhibitor. Consistent with *in vitro* data, macroautophagy was induced in the cortex and hippocampus of mouse brains treated with methylene blue. Our findings suggest that methylene blue-induced neuroprotection is mediated, at least in part, by macroautophagy though activation of AMPK signaling.

## INTRODUCTION

Methylene blue has long been used as treatment for methemoglobinemia (Dotsch et al., 2000; Zenk, 2001; Matisoff and Panni, 2006), ifosfamide neurotoxicity (Raj et al., 2004; Patel, 2006; Richards et al., 2011), malaria (Rengelshausen et al., 2004; Zoungrana et al., 2008; Coulibaly et al., 2009; Krafts et al., 2012). Recent studies suggest that methylene blue plays a critical role in neuroprotection against ischemic stroke and Parkinson’s disease due to its capability as an alternative mitochondria electron carrier (Wen et al., 2011). A very recent study indicates that methylene blue reduced Tau protein aggregates in Alzheimer’s disease through inducing macroautophagy (Congdon et al., 2012).

Macroautophagy is one of the main mechanisms for maintaining cellular homeostasis, including for maintaining homeostasis and functions of central nervous system (Komatsu et al., 2006; Xilouri and Stefanis, 2010; Marino et al., 2011). In the soma, macroautophagy occurs to maintain normal cellular homeostasis. In the axons, axotomy and excitotoxic insult trigger the accumulation of autophagosomes in dystrophic axonal swellings (Yue et al., 2008, 2009). In addition, studies also show that macroautophagy plays a significant role against the progression of neurodegenerative diseases, including Huntington’s (Heng et al., 2010; Tsvetkov et al., 2010), Alzheimer’s (Ling and Salvaterra, 2009; Wong et al., 2011), and Parkinson’s disease (Yang and Mao, 2010; Bae et al., 2011; Wong et al., 2011; Lynch-Day et al., 2012). However, whether macroautophagy will play an important role in methylene blue-mediated neuroprotection is largely unexplored.

In this study, we evaluated the involvement of macroautophagy in the methylene blue-mediated neuroprotection. Our aim was to elucidate the mechanisms by which methylene blue protects neurons during nutrient insufficiency.

## MATERIALS AND METHODS

### EXPERIMENTAL ANIMALS

C57BL/6 mice (male, 8~12week old) were purchased from the Jackson Laboratory. All animal procedures were approved by the University of North Texas Health Science Center Animal Care and Use Committee. All experiments were conducted in compliance with institutional guidelines and NIH Guidelines for the Use of Animals in Neuroscience and Behavioral research. Twelve mice (*n* = 4 in each group) were intragastrically administrated 50 (low dose) or 100mg/kg (high dose) methylene blue (American Regent, Shirley, NY, USA) daily for two consecutive weeks. Then, mice were euthanized and brains collected. Cortex, hippocampus and subventricular zone (SVZ) were isolated, snip frozen in liquid nitrogen and stored in –80°C for further analysis.

### CELL CULTURE

Mouse HT22 hippocampal cell line was cultured in Dulbecco’s Modified Eagle Medium (DMEM) (Gibco) supplemented with 10% fetal calf serum (Hyclone), 50IU/ml penicillin and 50 μg/ml streptomycin (Invitrogen) in a humidified incubator with 5% CO_2 _ at 37°C. Cells at passage 8~12 were adjusted to 1.5×10^5^/ml and were plated in 12-well or 6-well cell culture plates (Corning). At 4 to 6 h after seeding, adherent cells were rinsed with phosphate buffered saline (PBS) twice and serum-free DMEM in the presence or absence of methylene blue was added for indicated time periods of culture. For methylene blue pretreatment and cell viability assay, cells were seeded into 96-well plates and cultured for 24 h in methylene blue-containing DMEM supplemented with 10% fetal calf serum, then followed by 12-h culture in serum-free DMEM. Cell viability was determined using Calcein-AM (Invitrogen) assay according to the manufacture’s instruction.

### FLOW CYTOMETRY

Floating cells and adherent cells were collected by gently flushing the wells with 2 mM EDTA-PBS. Cells were then centrifuged and stained with propidium iodide (BD Biosciences, cat# 556463) and Annexin V (BD Biosciences) according to the manufacture’s instruction.

### IMMUNOCHEMICAL STAINING

Cells were seeded in each well of 8-well Lab-Tek^TM^ chamber slides (Thermo Scientific) followed by methylene blue treatment as described above. Cells were then fixed with 4% paraformaldehyde and stained with a rabbit anti-LC3B antibody (Cell signaling) followed by staining with Alexa Fluor 594-conjugated goat anti rabbit IgG (Invitrogen). Fluorescent microscopy was conducted using an Axio Observer Z1 fluorescent microscope (Zeiss).

### WESTERN BLOT ANALYSIS

Cell lysate and mouse brain tissue lysate were prepared by homogenization in RIPA buffer (20 mM Tris-HCl, pH 7.5, 150 mM NaCl, 1 mM Na2EDTA, 1 mM EGTA, 1% NP-40, 1% sodium deoxycholate) and lysis on ice for 30 min. Protein amount was quantified using Pierce 660 nm Protein Assay (Thermo Scientific) following the manufacture’s instruction. Total protein from each sample was loaded onto 8% (for acetyl-CoA carboxylase (ACC) and mammalian target of rapamycin (mTOR) or 10% (for extracellular signal-regulated kinase (Erk), Akt, 5′ adenosine monophosphate-activated protein kinases (AMPKs), B-cell lymphoma 2 (Bcl-2), and β-actin) SDS-PAGE gel and electrophoresis was performed. For LC3B, proteins were loaded onto 4–20% Mini-PROTEAN® TGX^TM^ Precast Gels (Bio-Rad). Proteins were then transferred onto nitrocellulose membranes and blocked with 5% non-fat dry milk-PBS for 1 h at room temperature (RT). After three washes with 0.05% Tween-20-PBS, membranes were incubated with the following primary antibody reagents overnight at 4°C, respectively: (1) Rabbit monoclonal anti-AMPK, anti-Phospho-AMPK, anti-ACC and anti-Phospho-ACC (Cell Signaling); (2) Rabbit monoclonal anti-mTOR and anti-Phospho-mTOR (Cell Signaling); (3) Rabbit monoclonal anti-Erk1/2 (Cell Signaling) and anti-Phospho-Erk1/2 Thr202/Tyr204 (Cell Signaling); (4) mouse monoclonal anti-Bcl-2 (Santa Cruz); (5) rabbit polyclonal anti-Akt (Santa Cruz) and anti-Phospho-Akt1 (Thr 308; Santa Cruz), and (6) mouse monoclonal anti-β-actin (Santa Cruz). Membranes were washed three times with PBST and then incubated with horseradish peroxidase-conjugated secondary antibodies for 1 h at RT. Membranes were developed with SuperSignal West Pico Chemiluminescent Substrate (Thermo Scientific) and were visualized and the optical density of the identified protein bands on membranes was analyzed using a Biospectrum 500 imaging system (UVP, LLC).

### STATISTICAL ANALYSIS

Data were analyzed and results were presented as mean ± standard deviation. Student’s *t* test or one-way ANOVA was used for comparison of mean between the groups, and *p* < 0.05 was considered significant.

## RESULTS

### METHYLENE BLUE PROTECTS HT22 CELLS FROM SERUM DEPRIVATION

**FIGURE 1:**
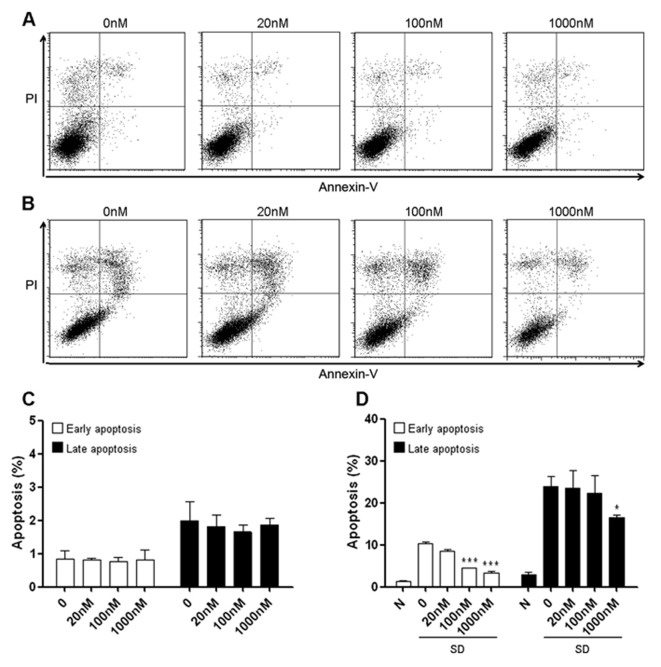
**Methylene blue attenuates serum deprivation-induced HT22 cell death.** **(A,C)** Methylene blue itself did not induce HT22 cell apoptosis under normal culture condition. **(B,D)** Methylene blue treatment significantly attenuated serum deprivation-induced apoptosis in a dose-dependent manner. *, *p* < 0.05; ***, *p* < 0.001. N, normal culture condition; SD, serum deprivation. *n* = 3 in each group, each study has been repeated at least three times.

We firstly tested whether methylene blue exerted cytotoxicity in cells under normal culture condition. Mouse hippocampal HT22 cells treated with methylene blue ranging from 20 nM to 1 μM for 24 h did not increase either early or late apoptosis, suggesting that methylene blue has no significant pro-apoptotic effect within this range of concentration (**Figures 1A, C**). Methylene blue did not reduce HT22 spontaneous apoptosis, compared with untreated control group (**Figures 1A, C**). As predicted, serum deprivation for 24 h robustly increased both early and late apoptosis (**Figures 1B, D**), which could be significantly inhibited by methylene blue in a dose-dependent manner (**Figures 1B, D**).

### METHYLENE BLUE INDUCES MACROAUTOPHAGY IN HT22 CELLS

**FIGURE 2:**
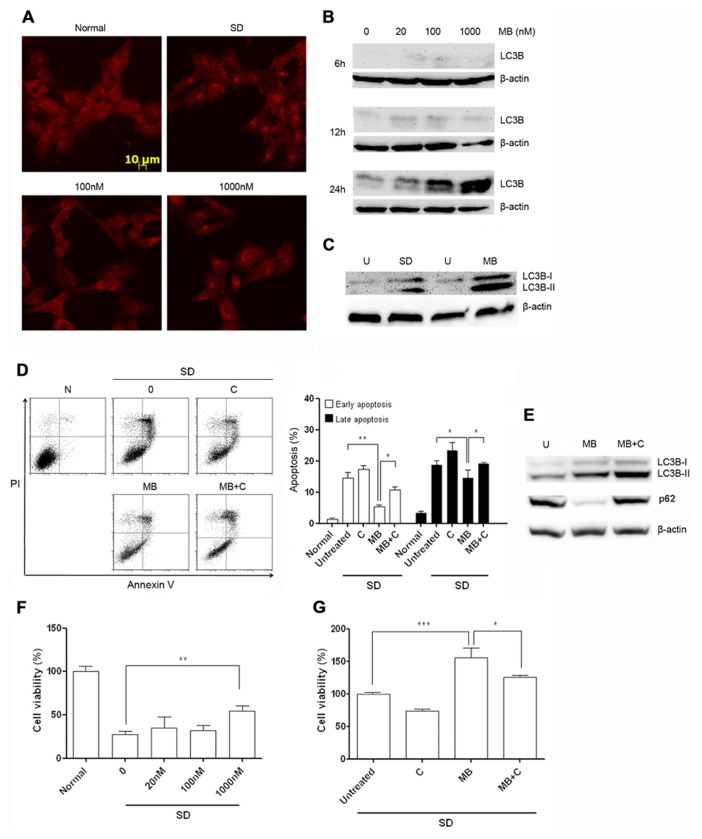
**Methylene blue induces macroautophagy in HT22 cells.** **(A)** Immunocytochemical staining of LC3B showed perinuclear aggregation of LC3B after 24 h treatment of methylene blue. **(B)** Representative Western blots showed that methylene blue increased both levels of LC3B-I and LC3B-II in a dose- and time-dependent manner. **(C)** Compared with 6-h serum deprivation, methylene blue robustly increased LC3B-II level. **(D)** Flow cytometry analysis indicated that chloroquine partially reversed methylene blue-induced anti-apoptosis after serum deprivation. **(E)** Western blot showed chloroquine increased LC3B-II level and stabilized p62 level in methylene blue-treated cells, suggesting chloroquine inhibited methylene blue-induced macroautophagy. **(F,G)** Calcein-AM assay demonstrated that methylene blue pretreatment significantly enhanced cell viability after serum deprivation which was blocked by chloroquine. *, *p* < 0.05; **, *p* < 0.01. N, normal; U, untreated; SD, serum deprivation; MB, methylene blue; C, chloroquine. *n* = 3 in each group, each study has been repeated at least three time.

To determine whether methylene blue-induced macroautophagy, HT22 cells were treated with methylene blue at the dose range between 20 nM and 1 μM and macroautophagy was then detected with immunocytochemical staining and Western Blot. Serum deprivation was set as the positive control for macroautophagy. Anti-LC3B antibody staining displayed a relatively homogenous distribution of LC3B in HT22 cells, which was predominantly located in the cytoplasma, consistent with other reports (Steiger-Barraissoul and Rami, 2009). Notably, perinuclear location of LC3B was observed after treatment of 20 nM or higher concentrations of methylene blue. In cells treated with 1 μM methylene blue, significant increased vesicular structure with strong LC3B staining in the perinuclear region, a specific morphological marker of promoted macroautophagy, was visualized, suggesting recruitment of LC3B into autolysosomes (**Figure 2A**). Western blot results showed a dose-and-time-dependent increase of both LC3B-I and LC3B-II in methylene blue-treated cells, with the most robust increase being induced by 1 μM methylene blue (**Figure 2B**). LC3B type II derives from processed LC3B type I and correlates well with autophagosome. Moreover, LC3B type II expression was increased in methylene blue-treated group, compared with serum deprivation group (**Figure 2C**). Collectively, these data suggested methylene blue is an efficient macroautophagy inducer in HT22 cells.

### METHYLENE BLUE PROTECTS HT22 CELLS THROUGH MACROAUTOPHAGY

To test the role of macroautophagy in methylene blue-mediated neuroprotection, HT22 cells were simultaneously treated with methylene blue and serum deprivation for 24 h, and cell apoptosis were determined by flow cytometry. Consistently, methylene blue rescued HT22 cells from serum deprivation-induced apoptosis. However, the recuing effect was significantly blocked in the presence of a potent macroautophagy inhibitor-10 μM chloroquine (**Figure 2D**). To confirm that chloroquine inhibited methylene blue-induced macroautophagy, we tested both LC3B conversion and p62 level in treated cells. It is well accepted that in cells undergoing autophagic process, LC3B-II level is increased, while p62 level is reduced due to its role in targeting proteins into autolysosome. The mechanism underlying the inhibitory effects of chloroquine on macroautophagy is to impairing lysosomal acidification, thus protein degradation in autolysosome is blocked. Our data showed methylene blue induced an increased LC3B-II level as well as a decreased p62 level in HT22 cells in comparison with the untreated cells, indicating methylene blue indeed activates macroautophagy. In the presence of chloroquine, LC3B-II level was even higher than that in cells treated with methylene blue alone, along with a stabilized p62 level, suggesting chloroquine indeed inhibits protein degradation in autolysosomes (**Figure 2E**).

Then, we tested whether methylene blue pre-treatment had protective effects. Compared with vehicle-treated group, methylene blue-pretreated HT22 cells were more resistant to apoptosis induced by serum deprivation, which could be blocked by chloroquine (**Figures 2F, G**). Chloroquine alone did not result in HT22 cell apoptosis. Collectively, these data suggested that macroautophagy play a critical role in methylene blue-mediated neuroprotection against serum-deprivation.

### mTOR SIGNALING WAS NOT INVOLVED IN METHYLENE BLUE-INDUCED MACROAUTOPHAGY

**FIGURE 3:**
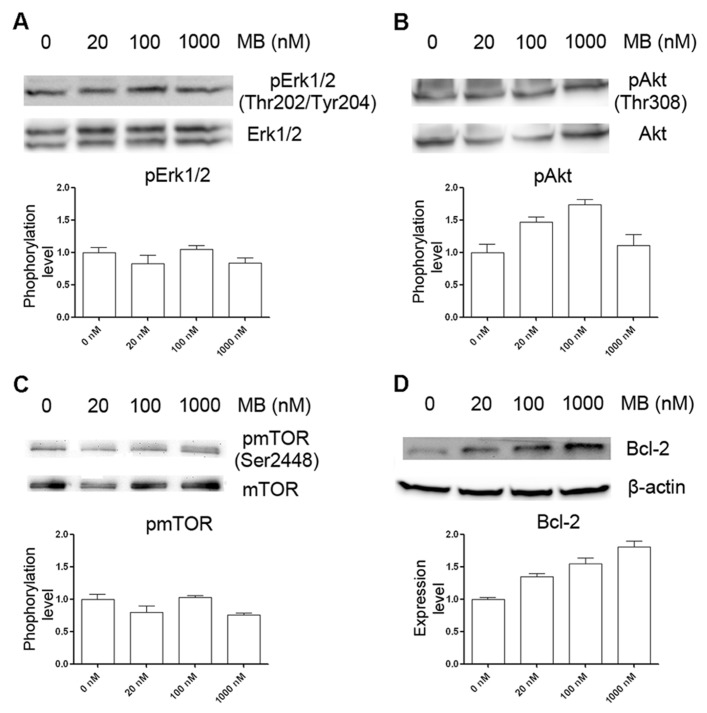
**Induction of macroautophagy in HT22 cells by methylene blue is not through mTOR signaling pathway.** **(A–C)** Representative Western blots showed that methylene blue did not alter activation of Erk1/2, Akt, or mTOR. **(D)** Representative Western bots depict that Bcl-2 protein level was increased by methylene blue in a dose-dependent manner.

To determine the signal pathways involved in methylene blue-induced macroautophagy, HT22 cells were treated with methylene blue under normal culture condition and Western blots were performed using anti-Akt and anti-Erk antibodies. As shown in **Figures 3A, B**, no significant change of activating phosphorylation of Erk1 or Akt was found in methylene blue-treated cells, compared with vehicle-treated cells. Although mTOR signaling has been suggested to be involved in macroautophagy in many systems, we found that phosphorylation of mTOR was not significantly changed upon methylene blue treatment at 100 nM or 1 μM (**Figure 3C**). Interestingly, the expression level of Bcl-2, a pro-survival and anti-apoptotic oncogenic protein, was robustly increased after methylene blue treatment in a dose-dependent manner (**Figure 3D**).

### ACTIVATION OF AMPK PATHWAY IN METHYLENE BLUE-INDUCES MACROAUTOPHAGY

**FIGURE 4:**
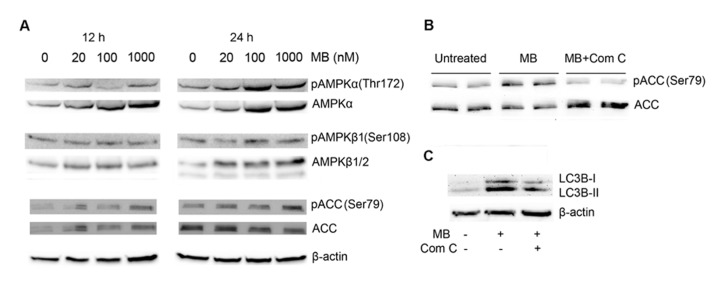
**Induction of macroautophagy in HT22 cells by methylene blue is mediated by AMPK pathway.** **(A)** Representative Western blots showed that methylene blue increased AMPKα, pAMPKα, AMPKβ1, and pAMPKβ1 in a dose-dependent manner. Activating phosphorylation of ACC was also enhanced. **(B,C)** Representative Western blots demonstrated that 20 μM Compound C (Com C) significantly reduced methylene blue-induced ACC activation and LC3B activation in HT22 cells. Data are representative of three independent experiments.

5′ Adenosine monophosphate-activated protein kinase pathway has been characterized as another signal pathway that regulates macroautophagy (Mihaylova and Shaw, 2011), we determined whether AMPK pathway was involved in methylene blue-induced macroautophagy. Compared with control, AMPKα and phosphorylated AMPKα were significantly increased at 12 and 24 h after methylene blue treatment in a dose-dependent manner. Similarly, AMPKβ expression was also increased. In addition, ACC was significantly activated at 24 h, confirming activation of the AMPK pathway (**Figure 4A**). To test whether AMPK pathway was critical for methylene blue-induced macroautophagy, cells were treated with 20 μM compound C, a selective AMPK inhibitor during the last 4 h of methylene blue treatment. Compound C co-treatment effectively inhibited methylene blue induced ACC phosphorylation, indicating that AMPK pathway was blocked (**Figure 4B**). Moreover, LC3B type II level was significantly alleviated by compound C, suggesting that methylene blue-induced macroautophagy was indeed via AMPK pathway (**Figure 4C**).

### METHYLENE BLUE INDUCED MACROAUTOPHAGY *IN VIVO*

**FIGURE 5:**
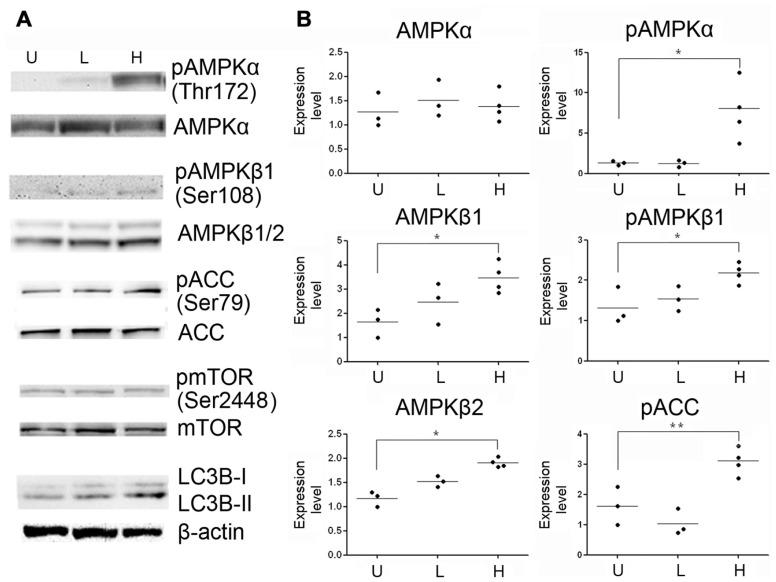
**Methylene blue induces macroautophagy in mouse hippocampus through AMPK signaling pathway.** **(A)** Representative Western blots depicts that phosphorylation of AMPKα, AMPKβ1 and ACC were enhanced in methylene blue-treated groups. Conversion of LC3B-I to LC3B-II was increased in methylene blue-treated groups. However, mTOR phosphorylation was not altered, compared with control group. **(B)** Quantitative analysis showed that methylene blue increase protein levels of AMPKβ1 and AMPKβ2. *, *p* < 0.05; ***, *p* < 0.001. U, vehicle control; L, low dose methylene blue; H, high dose methylene blue.

**FIGURE 6:**
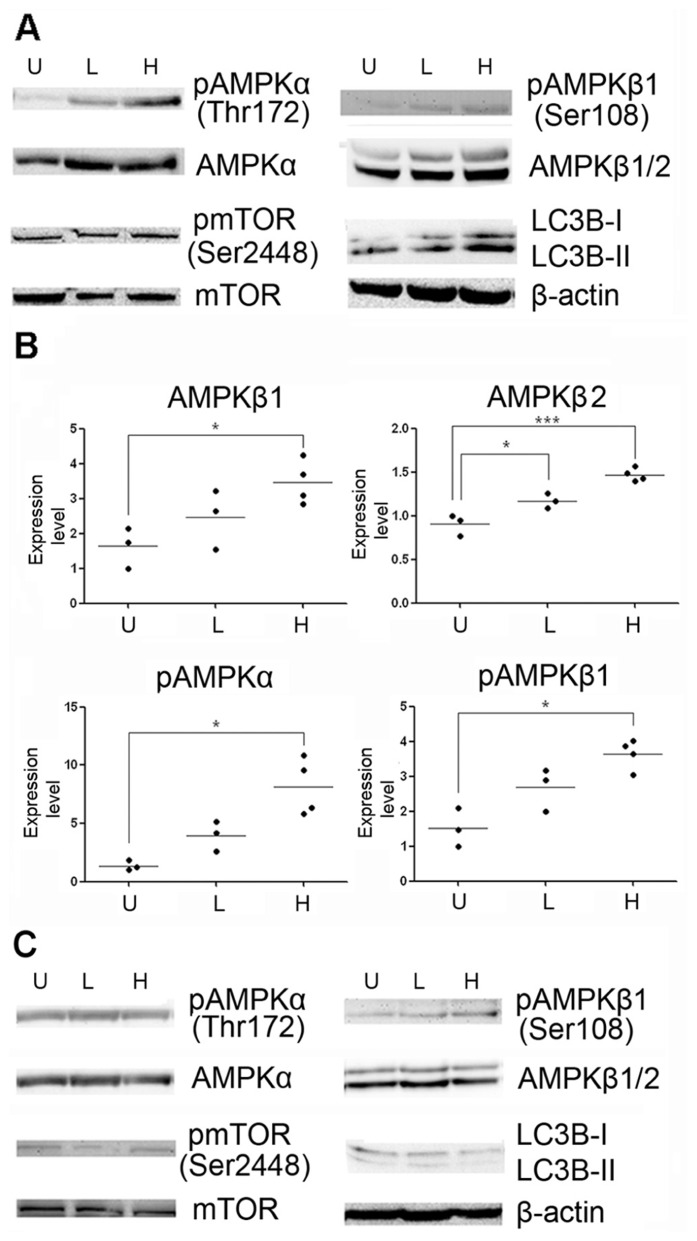
**Methylene blue induces activation of AMPK pathway in mouse brain.** **(A,B)** Representative Western blots and quantitative analysis showed that protein levels of AMPKα, AMPKβ1 and AMPKβ2, but not mTOR signaling, were increased at cortex in methylene blue-treated groups. Methylene blue did not significantly induce AMPK signaling activation in SVZ **(C)** *, *p* < 0.05; **, *p* < 0.01. U, vehicle control; L, low dose methylene blue; H, high dose methylene blue.

To determine whether methylene blue induces macroautophagy *in vivo*, we treated mice with methylene blue for 2 weeks, and AMPK activation and LC3B type II conversion in the hippocampus and cortex were then examined. As shown in **Figures 5** and **6**, no significant change of AMPKα protein level in the hippocampus and cortex was found upon methylene blue treatment. However, AMPKα phosphorylation was dramatically enhanced by methylene blue, especially in high dose-treated group. AMPKβ1 and AMPKβ2 were also significantly increased after methylene blue treatment in a dose-dependent manner. ACC phosphorylation was promoted in high dose group. Similar to *in vitro* data, phosphorylation of mTOR was not significantly altered in either methylene blue-treated group. Compared with vehicle-treated group, LC3B type II level was dramatically elevated, suggesting enhanced autophagosome formation in methylene blue-treated groups. In the SVZ, however, none of these signal pathways was altered after methylene blue treatment, indicating that the methylene blue might not affect neural stem/progenitor cells (**Figure 6C**). LC3B conversion also showed differences in distinct brain areas. Methylene blue increased LC3B type II in hippocampi and cortex while slightly decreased LC3B type II in SVZ of methylene blue-treated mice. Taken together, these data demonstrated that methylene blue can induce macroautophagy in hippocampus and cortex through AMPK but not mTOR pathway *in vivo*.

## DISCUSSION

In this study we find that (1) Methylene blue protects HT22 cells from serum deprivation-induced apoptosis through induction of macroautophagy; (2) Methylene blue induces macroautophagy via enhancement of AMPK pathway rather than inhibition of mTOR pathway *in vitro*; (3) Methylene blue induces macroautophagy via enhancement of AMPK signal pathway in mouse hippocampus and cortex *in vivo*; (4) Methylene blue does not induce macroautophagy in neural stem/progenitor cells located at the mouse SVZ *in vivo*.

Neurons in central nervous system are sensitive to inadequate serum supply. Studies have demonstrated the morphological and metabolic characteristics of neurons after serum deprivation (LeBlanc, 1995; Bae et al., 2011; Lesort et al., 1997; Poser et al., 2003; Chauvier et al., 2005; Steiger-Barraissoul and Rami, 2009). In the current study, we extend our previous findings and demonstrated that methylene blue exerts neuroprotective effect against serum deprivation. Recentstudies suggest that macroautophagy is neuroprotective against apoptosis induced by serum deprivation (Steiger-Barraissoul and Rami, 2009). Under nutrient starvation or energy deprivation circumstances, macroautophagy is thought to protect cells via saving and recycling materials for maintaining the synthesis of vital macromolecules. Our data showed that methylene blue treatment effectively protected starved cells from apoptosis and induced macroautophagy. When macroautophagy was blocked by chloroquine, the methylene blue-mediated neuroprotection was partially inhibited, suggesting that macroautophagy is involved in the anti-apoptotic action of methylene blue.

Previous studies have indicated that mTOR-related signal transduction is the key regulator of macroautophagy (Levine and Kroemer, 2008; Rabinowitz and White, 2010). Other signal pathways such as Akt and MAPK/Erk1/2 influence macroautophagy indirectly through regulating mTOR pathway. A very recent study has suggested that methylene blue induces macroautophagy to attenuate tauopathy through inhibiting mTOR activation (Congdon et al., 2012). Our data demonstrated that methylene blue did not significantly activate mTOR signaling both *in vitro* and *in vivo*. Consistently, Akt and Erk signaling, which has been proven to activate mTOR pathway, were not inhibited after methylene blue treatment. Bcl-2 was suggested to inhibit Beclin1-dependent macroautophagy. Methylene blue robustly increased Bcl-2 protein levels. Therefore, we speculated that Bcl-2 is not involved in methylene blue-induced macroautophagy. Rather, the anti-apoptotic Bcl-2 might be another mechanism contributes to the neuroprotectiove action of methylene blue.

5′ Adenosine monophosphate-activated protein kinase plays as a master regulator of cellular energy homeostasis (Hardie et al., 2012). The kinase is activated in response to stresses that deplete cellular adenosine triphosphate (ATP) supplies such as low glucose, hypoxia, ischemia, and heat shock. AMPK activation positively regulates signal pathways that replenish cellular ATP supplies while negatively regulates several proteins central to ATP consuming processes. It has been demonstrated that AMPK signaling positively regulate macroautophagy by activating Ulk1 through phosphorylation of Ser317 and Ser777, or indirectly by inhibiting mTOR signaling (Mihaylova and Shaw, 2011). Our data indicated that methylene blue induces macroautophagy via, at partially, AMPK signaling. Both AMPKα and AMPKβ1/2 protein levels were increased *in vitro*, whereas the changing pattern was more complicated *in vivo*, depending on the brain regions. In the hippocampus and cortex, the action of methylene blue on macroautophagy was similar to that *in vitro*, while no effect of methylene blue was observed in SVZ cells. Since SVZ consists of primitive neural stem/progenitor cells, we speculated that methylene blue-induced AMPK activation mainly occurs in mature neurons. Elaborating studies have shown that methylene blue act as an alternative electron carrier in the mitochondrial electron transport chain to enhance ATP production while energy is in short. So it is unlikely that AMPK activation in methylene blue-treated cells was due to increased AMP/ATP ratio. Promoted ACC phosphorylation indicated AMPK pathway was indeed activated. Methylene blue-induced AMPK activation was accompanied by higher levels of LC3B type II, the activated form of LC3 participating autophagosome formation. Furthermore, when AMPK inhibitor was administrated with methylene blue, ACC phosphorylation was reverted to basal level together with reduced LC3B type II level.

Taken together, the present study suggests that methylene blue exert protective effect against serum deprivation, at least partly, through enhancing macroautophagy. In addition, our study demonstrated that activation of AMPK pathway, but not mTOR, is involved in the methylene blue-induced macroautophagy.

## Conflict of Interest Statement

The University of North Texas Health Science Center has filed a PCT patent application entitled “Compounds that enable alternative mitochondrial electron transfer.”
